# Liddle Syndrome in Association with Aortic Dissection

**DOI:** 10.7759/cureus.1225

**Published:** 2017-05-04

**Authors:** Aamer Abbass, Jason D'Souza, Sameen Khalid, FNU Asad-Ur-Rahman, Joseph Limback, Jeremy Burt, Rajesh Shah

**Affiliations:** 1 Internal Medicine Residency, Florida Hospital-Orlando; 2 Internal Medicine, Florida Hospital-Orlando; 3 Diagnostic Radiology, Florida Hospital-Orlando; 4 Orlando Cardiac & Vascular Specialists, Florida Hospital-Orlando

**Keywords:** aortic dissection, liddle syndrome, premature hypertension

## Abstract

Liddle syndrome is a rare form of autosomal dominant monogenic hypertension manifested as an early onset of resistant hypertension with either no response or suboptimal response to conventional antihypertensive therapy. If there is a delay in diagnosis, uncontrolled hypertension can lead to end organ damage. To our knowledge, aortic dissection has not been reported in association with this disease. We report a case of a dissecting aortic aneurysm occurring in association with Liddle syndrome.​

## Introduction

Liddle syndrome (LS) is a rare form of monogenic hypertension first described by Sir Grant Liddle in 1963 [1]. It is perceived to be a very rare disease with a reported prevalence of < 1/1,000,000 at present [2]. Up until 2008, only 30 patients affected with this monogenetic disorder had been reported in the world [9]. Two small single-center studies have estimated the prevalence to be about 1.52% and 6% among hypertensive patients with genetic testing and phenotypical LS, respectively [3-4]. Our knowledge about LS is evolving, given its relative rarity. It typically manifests as early onset of resistant hypertension with either no response or suboptimal response to conventional antihypertensive therapy. Therefore, uncontrolled hypertension can lead to target organ dysfunction, such as stroke, heart failure, retinopathy, end-stage renal disease, etc. To our knowledge, aortic dissection has not been reported in association with this disease. We report a case of dissecting aortic aneurysm occurring in association with Liddle syndrome.

## Case presentation

**Figure 1:**
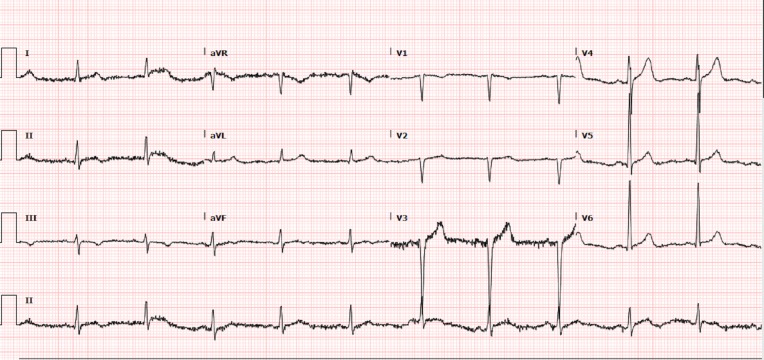
12-lead electrocardiogram with evidence of left ventricular hypertrophy

A 29-year-old Caucasian male with an established history of Liddle syndrome, diagnosed at the age of 8, and a 10-pack-year smoking history, presented to a local hospital with the sudden onset mid-sternal chest pain radiating to the back. The chest pain was associated with lightheadedness, headache, diaphoresis, palpitations, nausea, and one episode of non-bloody emesis. Family history was significant for LS in his father, younger brother, and paternal uncle. His father and paternal uncle met an early demise secondary to intracerebral bleeds from elevated blood pressure related to LS. In addition, he also confessed to being non-compliant with his antihypertensive medications for many years. His initial vitals were a temperature of 98.2 F, pulse rate of 83 beats/min, respiratory rate of 19 breaths/min, oxygen saturation of 98% on room air, and a blood pressure (BP) of 220/120 mmHg. His BP was equal in bilateral upper extremities with strong bilateral pedal pulses. A 12-lead electrocardiogram performed in emergency room revealed evidence of a left ventricular hypertrophy without any acute ST-T wave changes (Figure 1).

**Figure 2:**
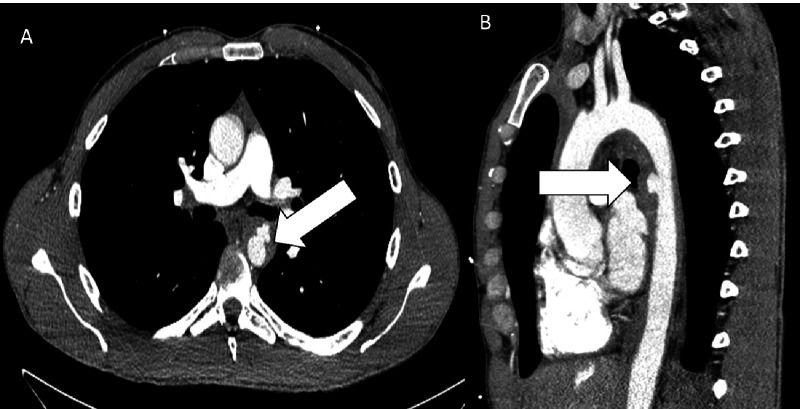
Computed tomography (CT) of the mid-chest A) Axial contrast-enhanced CT of the mid-chest showing dilation of the descending aorta with an intramural hematoma and penetrating ulcer (arrow) in the proximal descending aorta. B) Sagittal oblique CT showing the intramural hematoma and penetrating ulcer (arrow).

**Figure 3:**
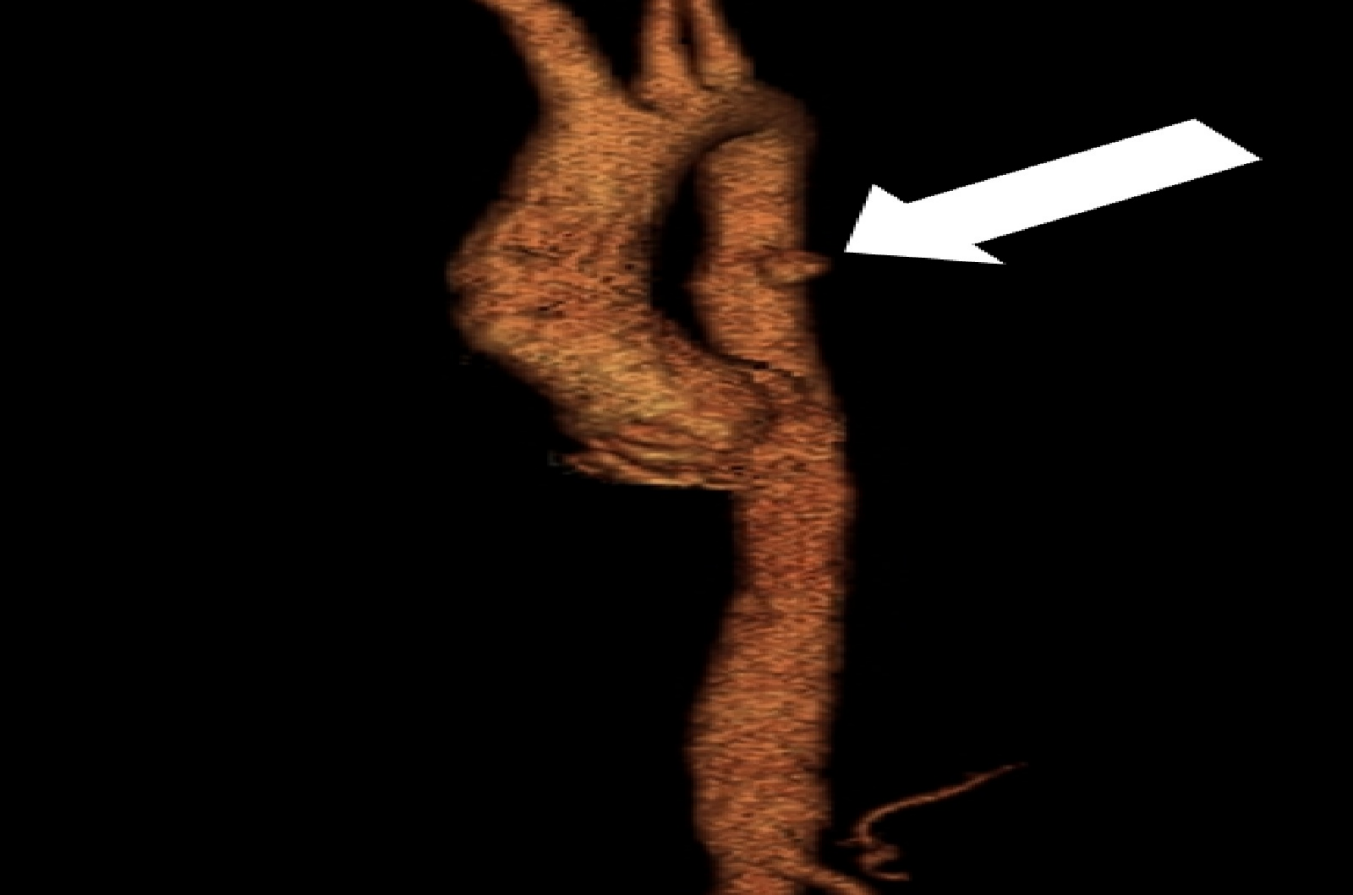
Volume-rendered computed tomography angiogram (CTA) demonstrating the penetrating ulcer (arrow).

**Figure 4:**
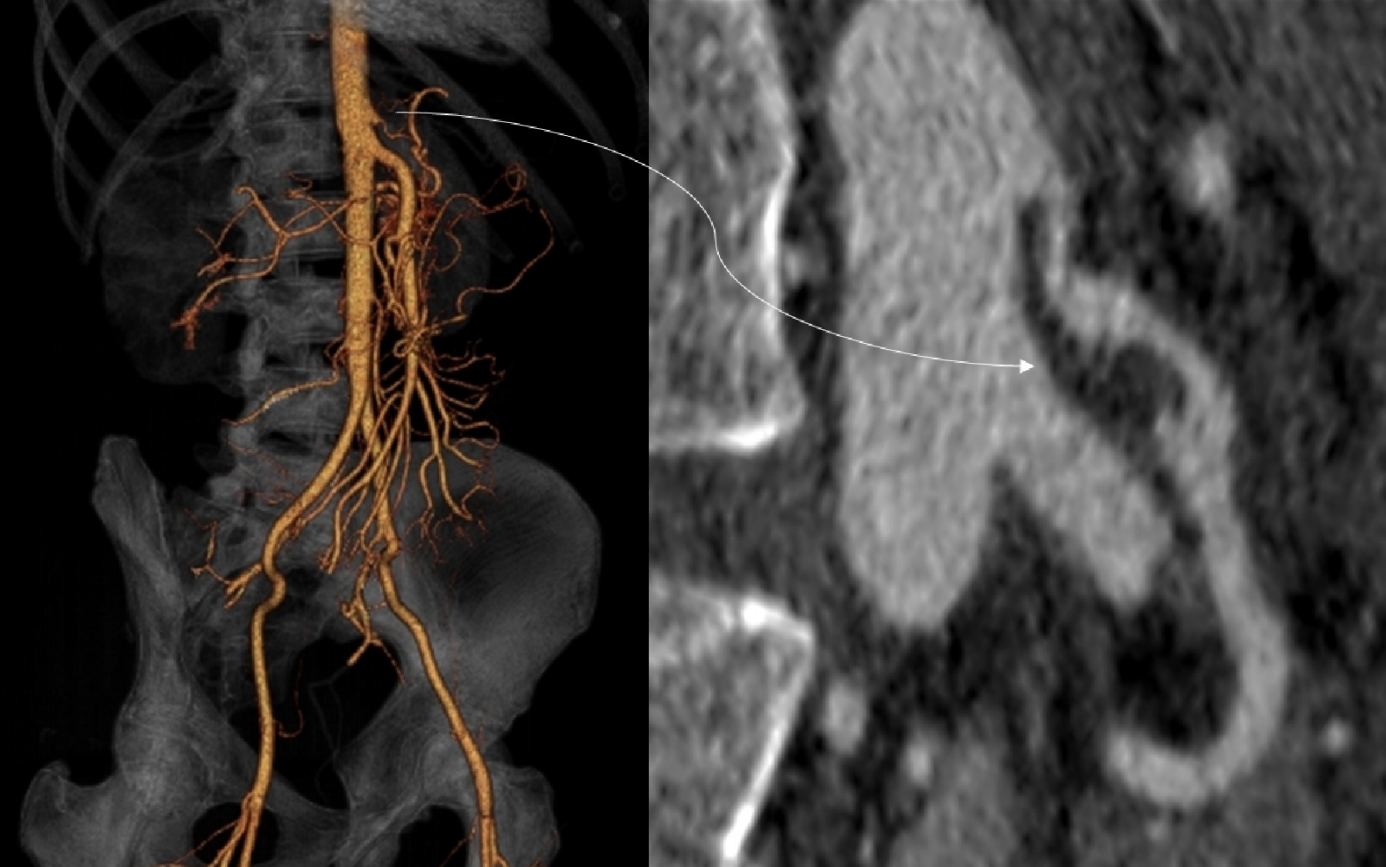
Volume-rendered and curviplanar reformatted computed tomography Images showing extension of the intramural hematoma into the celiac artery. This caused mild-moderate stenosis.

Given his concerning constellation of symptoms and findings on physical examination, a stat computerized tomographic angiogram (CTA) of the chest was performed, which showed an acute penetrating ulcer in the proximal descending thoracic aorta with intramural hematoma extending from the origin of the left subclavian artery into the suprarenal abdominal aorta, consistent with Stanford Type B aortic dissection (Figures 2-3). The intramural hematoma extended to the origin of the celiac artery resulting in a high-grade stenosis (Figure 4). CTA of the abdomen and pelvis also revealed fusiform dilatation of the suprarenal abdominal aorta with a maximal diameter of 3.4 x 3.3 cm.

The patient was immediately started on intravenous labetalol and nitroprusside drip for heart rate and blood pressure control and was subsequently transferred to our hospital for further management and hemodynamic monitoring. His initial lab work was notable for a sodium of 134 mEq/L, potassium of 3.9 mEq/L, chloride of 99 mEq/L, bicarbonate of 19 mEq/L, blood urea nitrogen (BUN) of 16 mg/dl, and creatinine of 0.84 mg/dl. Routine echocardiography demonstrated a normal ejection fraction of 60-65% with left ventricular hypertrophy. The patient required continuous intravenous infusions of various BP medications, including labetalol, nitroprusside, and nitroglycerin to achieve target BP goals in first 24-48 hours. However, it was not until the patient was started on 20 mg of oral amiloride that he was weaned off some of the anti-hypertensive drips. In addition, the patient also required clonidine, metoprolol, nifedipine, hydralazine, and isosorbide mononitrate to strictly maintain his BP at goal. By Day 3 of his hospitalization, BP control was achieved with an oral antihypertensive regimen as mentioned above and all the intravenous drips were discontinued. The patient remained asymptomatic during the ICU stay.

**Figure 5:**
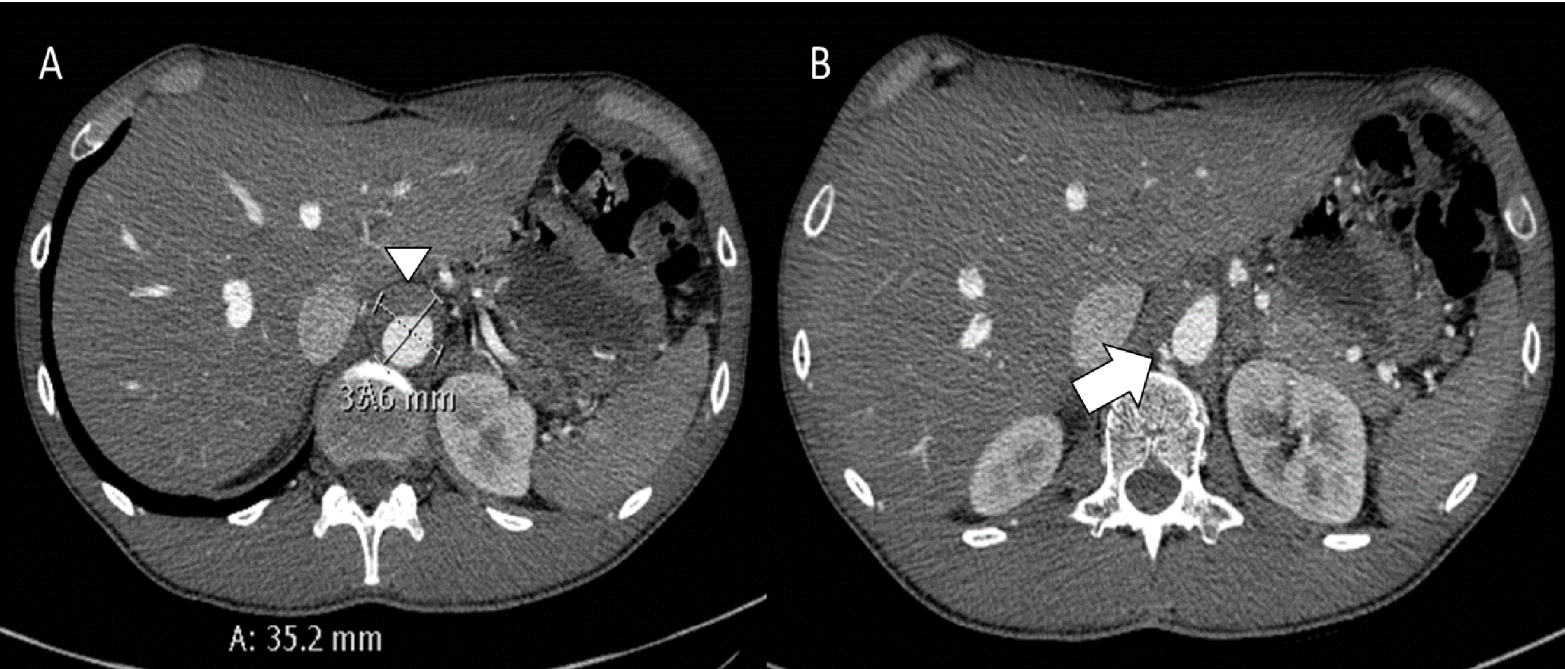
Axial contrast-enhanced computed tomography (CT) of the upper abdomen A) CT images showing an intramural hematoma with an aneurysm (arrowhead). B) Axial CT showing another small penetrating ulcer (white arrow).

A cardiothoracic consultation was obtained and medical management was the initial recommendation. However, on hospital Day 6, a repeat CTA of the chest, abdomen, and pelvis was performed to confirm stabilization of the penetrating ulcer. It revealed that the intramural hematoma in the mid-thoracic aorta had formed a deep penetrating ulcer, in addition to stable fusiform dilatation of the suprarenal abdominal aorta with a maximal diameter of 3.4 x 3.3 cm (Figure 5). 

Vascular surgery was consulted and the patient underwent thoracic endovascular aortic repair (TEVAR) with the exclusion of the intramural hematoma and penetrating ulcer in the mid-descending thoracic aorta using a Gore® TAG® Thoracic Endoprosthesis 26 x 10 (WL Gore & Associates, Inc., Flagstaff, AZ) on hospital Day 8. The patient had an uneventful recovery and was discharged on multiple oral BP medications as outlined above after 10 days of hospitalization. The patient followed up in the clinic one month after discharge from the hospital and reported feeling well with no further episodes of chest pain. His blood pressure had been well controlled on multiple oral antihypertensive medications, including amiloride.

## Discussion

Liddle syndrome is a rare form of hereditary monogenic hypertension. It is caused by a mutation of either β or γ subunits of SCCN1B and SCCN1G genes, which prevents degradation of the epithelial sodium channel (ENaC) of distal convoluted tubule from proteasomes, resulting in constitutive activation of the ENaC. This constitutive activation causes unregulated sodium and water reabsorption and volume expansion leading to uncontrolled hypertension [5]. The result of this cascade of events is a negative feedback of the hormonal axis leading to a low renin-low aldosterone state, and hence, the term pseudo-aldosteronism. Hypokalemia and metabolic alkalosis associated with this feedback mechanism is the norm rather than the rule and provides important diagnostic clues [6-7]. Therefore, normal serum chemistries, such as in our patient, can be expected.

Target organ dysfunction is not an uncommon complication, given the early age of onset and longstanding uncontrolled hypertension, as the condition is not readily diagnosed and treatment differs from essential hypertension. Under-recognition and inappropriate treatment can lead to sustained hypertension and associated complications, including (but not limited to) renal failure, hypertensive heart disease, cerebrovascular mortality, retinal damage, and, in severe cases, premature and sudden death prior to the age of 40 [8]. To the best of our knowledge, our patient is the index case of Liddle syndrome presenting with aortic dissection. Diagnostic clues include a positive family history, characteristic lab findings, the lack of response to conventional antihypertensives but a dramatic response to ENaC blockers, and genetic testing consistent with the underlying mutation. Differential diagnosis includes glucocorticoid-remediable aldosteronism, apparent mineralocorticoid excess, primary aldosteronism, Cushing’s syndrome, pheochromocytoma, renovascular hypertension, essential hypertension with diuretic use, and congenital adrenal hyperplasia. Treatment options are limited. However, most patients demonstrate a dramatic and remarkable response to a salt-restricted diet and ENaC blockers, such as amiloride and triamterene. Amiloride competes with sodium at the level of the ENaC conducting pore to prevent sodium reabsorption [9].

## Conclusions

Although readily treatable, Liddle syndrome is frequently underdiagnosed and widely misrecognized. Genetic testing is recommended for patients and first-degree relatives to uncover existing and de-novo mutations. Although LS is considered to be a rare condition, increased awareness and more frequent genetic testing may lead to increased reported prevalence.
